# Community Advisory Boards in Community-Based Participatory Research: A Synthesis of Best Processes

**Published:** 2011-04-15

**Authors:** Susan D. Newman, Jeannette O. Andrews, Gayenell S. Magwood, Carolyn Jenkins, Melissa J. Cox, Deborah C. Williamson

**Affiliations:** Medical University of South Carolina, College of Nursing, Charleston, South Carolina; Medical University of South Carolina, College of Nursing, Charleston, South Carolina; Medical University of South Carolina, College of Nursing, Charleston, South Carolina; Medical University of South Carolina, College of Nursing, Charleston, South Carolina; Medical University of South Carolina, College of Nursing, Charleston, South Carolina

## Abstract

Community-based participatory research (CBPR) is a paradigm to study and reduce disparities in health outcomes related to chronic disease. Community advisory boards (CABs) commonly formalize the academic–community partnerships that guide CBPR by providing a mechanism for community members to have representation in research activities. Researchers and funding agencies increasingly recognize the value of the community's contribution to research and acknowledge that community advisory boards are a key component of successful CBPR projects. In this article, we describe the best processes for forming, operating, and maintaining CABs for CBPR. We synthesize the literature and offer our professional experiences to guide formation, operation, and maintenance of CABs.

## Introduction

Community advisory boards (CABs) often serve as a source of leadership in the partnerships of community-based participatory research (CBPR) and provide structure to guide the partnership's activities. CAB composition typically reflects the community of interest; its members may share a common interest, identity, illness experience, history, language, or culture (1). CABs provide an infrastructure for community members to voice concerns and priorities that otherwise might not enter into the researchers' agenda, and advise about suitable research processes that are respectful of and acceptable to the community (2). Research assessing the roles, responsibilities, and processes of CABs supports their effectiveness in building mutually beneficial partnerships between academic researchers and communities (3-7). However, not all community-based researchers have incorporated CABs, nor have CABs been successful in every setting or situation (8,9).

The Center for Community Health Partnerships at the Medical University of South Carolina (MUSC) is a group of community partners, researchers, clinicians, and educators whose purpose is to engage and mobilize academic–community partnerships that promote health and lessen the impact of chronic illness (10). The Center provides a systems-level infrastructure for MUSC academic–community partnerships and promotes institutionalization and sustainability of these partnerships and their products. The Center's founding members formed a CAB to guide its vision and mission. This process prompted a review of the literature and discussions about the purpose of the board, membership, operating procedures and principles, leadership roles, training needs, sustainability, and evaluation. Our immediate goal was to identify the best processes for forming, operating, and maintaining a CAB. To accomplish this goal, we adopted the integrative practice framework from Cargo and Mercer, which identifies a continuum of CBPR processes from initial engagement to maintenance (11). We based the concept of best processes on Green's recommendations that academic–community partnerships tailor established processes to meet their unique needs (12). A central issue in the adoption of these processes is the transfer of knowledge to the practitioners in the field, whether academic or community, and to recognize the multiple factors that influence adoption and implementation of these processes in all settings and stages (13). In this article we present *best processes* for forming, operating, and maintaining CABs that guide CBPR, by synthesizing processes reported in the literature and demonstrating their adoption and implementation in the field using exemplars from our Center members' experiences.

Two of the Center's academic researchers (S.D.N., J.O.A.) conducted a review of the literature to identify processes of CAB functioning. We searched Ovid/Medline, CINAHL, and PsycINFO databases for manuscripts published in English from 2000 to 2009 by using the following search terms: "community advisory boards," "advisory boards," or "community steering committees," and "community-based participatory research" or "participatory research." Inclusion criteria were descriptions of CABs, which included in-depth discussion of roles, purpose, and structure in guiding community research. Our search revealed few published, peer-reviewed articles that focused solely on the development and functioning of a CAB (2,4,5-7,9,14-16). Rather, we found discussions of CABs embedded in articles discussing CBPR, often making this valuable information difficult to find through traditional search strategies. Additionally, bibliographies provided a rich resource for other publications and sources that described CABs. Additional searches were conducted in CBPR textbooks (17-19) and other CBPR-related documents, such as websites and listserves (20).

During our analysis and synthesis of the literature, we identified key processes of CAB functioning and coded our findings in an organizational matrix with 3 domains (formation, operations, maintenance) on the basis of an adaptation of Cargo and Mercer's framework (11). We then solicited input from Center members (G.S.M., C.J., M.J.C., D.C.W.) who had experience with CABs and requested that they review the matrix and reflect on best processes on the basis of their experiences. We held team meetings to cross-check the literature synthesis and personal experiences, reconcile analyses to identify processes for each domain of the matrix, then refine description of the processes on the basis of discussion and consensus. We quickly determined that the processes of CAB functioning are not linear but are iterative and cyclical, and may overlap or be revisited. We presented the initial findings at a national conference of academic CBPR researchers and to the Center's academic and community representatives to further validate the findings. We held subsequent team discussions to refine the findings on the basis of feedback we received.

## Overview of Research at the Center for Community Health Partnerships

The Center houses 45 projects with approximately $6.5 million annual expenditures. The projects involve partnerships with various communities and are at various stages in partnership development and research implementation. Approximately half of the projects have study-related CABs. We will describe the Center's overall CAB and 3 study-related CABs (Appendix A). All studies received approval from the MUSC institutional review board.

The Center's 20-member CAB is composed of representatives from regional for-profit, nonprofit, school, faith-based, and government organizations, as well as community members. The purposes of the Center's CAB are to 1) identify community priorities, needs, and interests; 2) set research priorities; 3) provide input or resources or both for the Center's research activities; 4) identify community members to participate on project steering committees; and 5) promote community support for and involvement with research.

### Partnership with people with spinal cord injury (Photovoice)

The Photovoice study (21,22) aimed to identify and address barriers and supports to community participation for people who use wheelchairs for mobility and was the catalyst for the formation of a CAB representing their interests.

Wallerstein and Duran contend that the best CBPR practices require an emancipatory perspective that promotes the participation of community members to transform their lives (23). People with disabilities have expressed a need for inclusive, action-based research methods in which they function as partners and consultants, not as research subjects (24-26). Our 6-member CAB is composed of people with spinal cord injury and the director of a nonprofit disability advocacy organization. People who participated in the Photovoice project and expressed an interest in continuing their role as a partner in research agreed to create a more formalized CAB. This CAB continues to serve as a partner with the academic researcher (S.D.N.) and share decision-making power regarding conduct of research and use and ownership of the products.

### Partnership with public housing residents (Sister-to-Sister)

In 2001, an inner-city school official invited the academic investigator (J.O.A.) to work with the community to help women and families to quit smoking (27,28). The academic and community partners agreed to form a 5-member working group of local laypersons ("insiders") to provide guidance on community preferences, contexts, and a comprehensive community assessment. The following year, on the basis of community interest and initial compatibility of the project, an 8-member CAB was formed, consisting of housing authority officials, members of for-profit and nonprofit community organizations, and lay community members. The purpose of the CAB is to guide the development, implementation, and evaluation of a smoking cessation intervention tailored for women (ie, Sister-to-Sister) living in public housing neighborhoods. After several feasibility and pilot studies, this collaborative partnership is now engaged in a randomized controlled trial that is testing the effectiveness of a multilevel smoking cessation intervention in public housing neighborhoods in 2 states. Because of the complexity, scope, and expansion of the study, neighborhood advisory boards in each of the intervention neighborhoods ensure that the intervention activities are relevant to each site.

### Partnership with coalition on diabetes (Charleston-Georgetown Diabetes Coalition)

In 1999, the Charleston-Georgetown Diabetes Coalition applied for a Centers for Disease Control and Prevention (CDC) Racial and Ethnic Approaches to Community Health (REACH) grant and asked the MUSC (C.J., G.S.M.) to lead the group's efforts (29-31). Each of the organizations or communities that are part of the coalition selected 1 representative to become a lead member of the coalition. The group has 10 funded partner members and 4 other members who are engaged in community activities in the 2-county area. Members are added by invitation of a coalition partner and approval by 70% of current members. All members work together to direct research and support community efforts related to diabetes in the African American community. Anyone from the community or local organizations may bring issues, concerns, suggestions, or requests to the group for action.

## Defining Processes for Formation, Operation, and Maintenance

CABs may engage in processes of formation, operation, and maintenance to accommodate the realities of working in a dynamic community setting (12). *Formation* processes address key activities related to defining the role and purpose of a CAB and subsequent identification and recruitment of key stakeholders from the community for participation in the CAB. *Operation* processes address the development of procedures to guide the logistical operation of the CAB, the development of guiding principles to assure the values of the community are represented and respected, and the establishment of leadership and decision-making protocols. *Maintenance* processes address evaluation of CAB actions and outcomes and plans for sustainability. Ongoing attention to evaluation and sustainability is essential to the maintenance of both newly formed and long-standing CABs. Results of evaluation assessments and strategic planning for sustainability may require CABs to address processes of formation and operations once again.

## Best Processes: Formation

### Clarifying purpose, functions, and roles

CBPR teams often form a CAB to gain representation of community perceptions, preferences, and priorities in the development of a research agenda and research processes (32). Examples of additional board functions include advising on study protocol design and implementation, facilitating community consent, evaluating and communicating the risks and benefits of research, helping provide resources, evaluating education materials, disseminating information, and using research findings to advocate for policy change (5,6,9,27,33).

Ideally, CAB members function as partners in CBPR; however, members are often placed in the role of advisors. "Partners" and "advisors" each operate at a different level in the partnership power gradient. Members in a *partnering role* bring issues and concerns from the community to the table, which the board discusses and resolves in a manner that is mutually beneficial to both the research team and the community (7). Members serving in an *advisory role* provide information, guidance, or suggestions from the community; however, the research team may choose to accept or reject the advisors' input (7). Clarification of both the intended purpose of the CAB and intended roles of CAB members facilitates the selection and recruitment of appropriate community representatives to serve on the board and maximizes their contribution as research partners. The members of our individual study CABs work in a partnering role with the academic partners, making collaborative decisions in their respective studies through each stage of the research process (Appendix A).

### Determining membership composition and recruitment strategies

To select appropriate board members, specific inclusion criteria should be established that reflect the goals of the research and the intended functions and purpose of the CAB (19). Brainstorming to identify potential members and determine the best recruitment and selection strategies is an iterative process requiring input from all members of the research team (32). The process requires consideration of types of expertise and resources needed and who can bring that expertise to the partnership. The intended outcomes of the study facilitate determining what type of person (eg, service provider, consumer, community leader) or agency is represented on the CAB (34). Identification of people or agencies with specific expertise in the topic of interest is necessary to create a knowledgeable CAB and to help position the research project favorably in the community. New partnerships are often encouraged to start small and to involve a few community-based organizations that are highly regarded by community members (35). The composition of the CAB for people with spinal cord injury increases consumer direction of disability and rehabilitation research. As the research program progresses, the CAB can decide whether to expand CAB membership by inviting service providers, agency leaders, and other community stakeholders to participate in an advisory or partnering role.

Our Center assesses community and capacity to guide identification of potential partners (36,37). Center organizers created a "potential member matrix" that includes the types of organizations to be considered; their reputation, activities, and achievements in the community; their capability to contribute resources; their self-interests; and their potential conflicts. The matrix facilitated preliminary fieldwork to identify potential CAB members (19). Once people or agencies meeting the initial inclusion criteria were identified, a process of screening (telephone and personal interview) and recruitment (personal invitations followed by letters to the organization) was used to refine the selection process, to carefully evaluate those who expressed an interest in serving, and to assure a good fit with the intent and purpose of the CAB.

Before gaining final commitment to serve, the CAB and potential member should review the potential member's intended role and clarify expectations, including and defining mechanisms of communication to help ensure a shared understanding of the requirements of the board member position. A signed letter of commitment provides documentation of the agreement and helps to minimize potential misunderstandings. The REACH Charleston-Georgetown Diabetes Coalition uses a document outlining the roles and scope of work for each partnering organization: the document is signed by both the partner representing the community organization and the academic partner and is renegotiated annually.

Generating a new CAB to work on a community issue may not always be the right approach or the best use of resources. Locating a CAB partnership in an existing community structure may be a more effective strategy; in such a situation, the academic partner asks for admission to the partnership and in turn forms a work group within the existing organization. Partnering with an existing group may also promote sustainability; however, this approach is not well described in the CAB literature and requires further examination to determine the benefits and pitfalls.

## Best Processes: Operation

### Establishing operating procedures

Operating procedures provide logistical guidance regarding how the team works together to complete tasks, including setting the agenda and documenting minutes. When establishing procedures, consideration of group dynamics and accepted social norms must be considered to ensure open communication (38). Procedures that address group dynamics include having everyone listen to one another and demonstrate mutual respect, letting members agree to disagree, having all members participate in board meetings and activities, and having meetings start and end on time (35,39). Members periodically reassess and revise the procedures, on the basis of process evaluations, to maintain an equitable balance of power (36) (Appendix B).

### Establishing operating principles

Defining the community values or principles that guide research is another initial task of a CAB (15). The process of developing principles that reflect the local context provides the opportunity to develop trust and build relationships among board members. The CAB then uses these principles to evaluate research protocols to assure that they honor and protect the values of the community (15). Resources (40,41) provide a framework on which a CAB can build principles that are specific to the context of its community and the research project. The CAB of the REACH Charleston-Georgetown Diabetes Coalition used the Community-Campus Partnerships framework to develop partnership principles (Appendix C).

### Establishing leadership, balancing power, and making decisions

A key element of effective group process is the fair and appropriate distribution of power and leadership; however, balancing power among diverse partners who represent multiple levels of social hierarchy is challenging (38). A potential strategy is to maintain community and academic cochairs; 2 community cochairs may lessen the possibility that academia dominates the community, especially in settings with a history of extreme power imbalance (32). The CAB for the Sister-to-Sister study uses a written protocol that clearly delineates the responsibilities of the partnership's cochairs (Appendix D). Effective leadership and balancing of power supports members' satisfaction, participation, and overall effectiveness by using democratic and consensus-based decision-making (19,42).

CABs generally find that the decision process runs more smoothly if they establish a protocol for decision-making. For example, a designated member may make low-stakes decisions independently, such as determining the typeface for a brochure (38). Having small subcommittees is an effective approach to making decisions on issues that do not require input from the entire CAB membership. Subcommittees decentralize decision making, help balance power, and provide the opportunity for partners, who may feel intimidated in large groups, to participate freely in small group discussions (38). Complex, high-stakes issues generally require a decision by consensus; however, gaining consensus does not mean that the decision must be unanimous (19). The 70% majority is a common strategy for meeting consensus that works well for the Sister-to-Sister CAB. Consensus decision making is often a more time-consuming process; however, incorporating everyone's opinions results in collective support by the CAB membership and increased group solidarity on the decision (19).

## Best Processes: Maintenance

### Evaluating partnership processes

A multimethod approach to collecting evaluation data increases the likelihood of a well-rounded assessment of the CAB structure and processes. Key informant interviews, meeting observations, focus groups, documents such as activity logs, and member surveys provide different perspectives of the partnership and enhance the comprehensiveness and credibility of evaluation (43). Qualitative methods, such as key informant interviews, provide a platform for CAB partners to address frustrations and concerns (44) (Appendix E).

Quantitative methods, such as surveys, provide a standardized measure of partnership processes that allows a baseline measure to be established and reevaluated over time to gauge continued effectiveness (45). Measures of process evaluation incorporate items to assess group dynamics within a CAB partnership framework, including shared leadership, open communication, mechanisms for resolving conflicts, and trust and cohesion (44,46,47). Evaluation of CAB leadership considers whether leaders provide praise and recognition, seek out members' opinions, and approach members for help with specific tasks (45). Process evaluation also includes assessment of more pragmatic issues such as turnover rate of board members, success in recruiting members with specific skills or connections to influential leaders, members' perceptions of the benefits and costs of participation, and the degree to which members perceive the partnership to be effective and sustainable over time (45,47). Evaluations that address partnership priorities increase the likelihood that partnership collaboration continues, thus promoting sustainability (19,43).

### Sustainability

A plan for sustainability is essential during the early stages of partnership. CAB functioning influences the survival of partnerships, because well-managed boards are often able to continue even amid funding difficulties (48). Formal sustainability planning ideally begins before initiation of research, but at a minimum of 1 year before the active project or current funding ends (49). The CAB defines the meaning of sustainability for the partnership and the criteria for sustainability that members will use to evaluate components of the partnership or program.

Strategies that instill a sense of empowerment and capacity building are essential to promote the retention and satisfaction of CAB members. Training in the principles of CBPR and the language and skills of research helps build the capacity of the CAB and generate belief in the partnership's ability to enact change in the community.

Recognition of CAB members' contributions of time, resources, and expertise, through some type of compensation, promotes continued engagement in the partnership (49). Many partnerships do not have the means to provide monetary remuneration. Identifying other means to promote member retention and ensuring that the benefits of membership outweigh the costs is essential. Such strategies may include adequate orientation and training of new members, opportunities for social interaction and participation, adequate access to information and resources, influence in decision making, and recognition for contributions (19). Inexpensive strategies to recognize members' contributions include potluck dinner parties, awards or honors given by the partnership, positive letters to a member's colleagues or superiors, and public recognition in local media (49).

Continuing relationships informally during gaps in funding or activities helps to maintain communication between partners and provides the opportunity for brainstorming about the next steps for the partnership. Gaps in funding also provide an opportunity to think ahead and plan for ways to avoid, or at least minimize, these gaps in the future. When sustainability is not possible, clear communication between the researchers, the CAB, and community members will leave the door open for future collaborations. The partnership developed in the Photovoice study has experienced gaps in funding yet remains viable and is currently engaged in another funded project.

## Conclusion

A CAB provides a focus for research efforts, an ongoing partnership to address community health concerns, and a mechanism for building capacity in the community and the academic institution. Establishing and sustaining a CAB is a time- and labor-intensive process — which many new partnerships underestimate. Careful initial consideration of the desired functions of a CAB will indicate whether the need is to create a new or expand an existing partnership to improve the health of the community. Continuing to share successes and challenges related to the processes of forming, operating, and maintaining effective CABs promotes ongoing learning and provides a frame of reference for continuing action and research on the best processes in CBPR.

## Appendices

### Appendix A. Activities and Decisions of Community Advisory Boards, by Study and Project Phase

**Photovoice (21,22)**

Identifying the problem

- Identifies environmental factors affecting community participation after spinal cord injury.

Study design and project startup

- Reviews and endorses application for funding.
- Allots study "work space" in agency facility.
- Obtains funding.

Participant recruitment and data collection methods

- Reviews and refines participant inclusion criteria and recruits participants.
- Discusses and approves participant incentives (eg, food at meetings, cameras).
- Identifies adaptive equipment (eg, cable release, tripods) and refines data collection protocol to minimize transportation issues.

Data collection, analysis, and interpretation

- Collects photographic data of community environmental factors.
- Provides interpretation of photos in 1-to-1 interviews with academic partner.
- Interprets results of collective group findings during celebratory meeting.
- Identifies key issues for action and strategizes next steps.

Dissemination

- Coauthors peer-reviewed manuscript reporting study process and outcomes.
- Designs and distributes pamphlet at Disability Advocacy Day.
- Organizes training in legislative advocacy.
- Engages local media (eg, newspaper).
- Engages state legislators for policy change.

Evaluation and reflection

- Identifies problems to be addressed in the subsequent project.
- Identifies potential future partners to expand the capacity of board.
- Identifies training needs to increase capacity for future community-based participatory research intervention studies.

**Sister-to-Sister (27,28)**

Identifying the problem

- Sponsors town hall meetings in community to determine interest.
- Codevelops quantitative survey for administration to a random sample of women in public housing neighborhoods.

Study design and project startup

- Guides intervention development based on survey of community women (ie, multilevel intervention).
- Negotiates study design (ie, delayed intervention in comparison neighborhoods).
- Reviews and approves all instruments.

Participant recruitment and data collection methods

- Determines incentives for participants (eg, gift cards, lotions, kitchen tools) and methods for recruitment.
- Community advisory board, community representatives, and hired community health workers participate in recruitment.
- Consensus on data collection methods and time frames.

Data collection, analysis, and interpretation

- Assists with evaluation of qualitative data.
- Assists with interpretation of quantitative and qualitative data.

Dissemination

- Creates community newsletter (quarterly dissemination).
- Holds neighborhood cookouts to disseminate major findings at end of pilots.
- Engages local media (eg, radio, newspaper).
- Coauthors scientific abstracts and publications.

Evaluation and reflection

- Evaluates board processes and products of research by using focus groups, key informant interviews, surveys, and advisory board meeting minutes data.

**REACH Charleston-Georgetown Diabetes Coalition (29-31)**

Identifying the problem

- Community partners join to form REACH Charleston-Georgetown Diabetes Partners Coalition.
- Identifies the community assets and needs related to diabetes for African Americans living in the 2 counties.

Study design and project startup

- Designs a comprehensive assets and needs assessment.
- Develops 3-pronged intervention approach: 1) community education and diabetes self-management training, 2) health systems change led by community partners and staff, and 3) coalition building for collaboration and community action.

Participant recruitment and data collection methods

- Works collaboratively as partners to decrease disparities.
- Hires and trains community health workers, registered dietitians, and registered nurses to recruit community members and volunteers.
- Collects and examines epidemiologic data and audits medical records related to diabetes.
- Conducts focus groups with community leaders, health professionals, and people with diabetes and their support networks.
- Conducts survey of people with diabetes.

Data collection, analysis, and interpretation

- Continues data collection and tracking the number of participants and community events by partners and staff.

Dissemination

- Participates in providing feedback to health agencies where audits occurred.
- Shares data with community groups through newsletter, quarterly written reports, news releases, and presentations.

Evaluation and reflection

- Assists with evaluation and action plan for each year: 1) annual medical records audit by staff with report and planning by partners; 2) annual focus groups with community leaders, health professionals, and people with diabetes and their support persons; and 3) annual survey of community residents.

### Appendix B. Example: Operating Procedures — Racial and Ethnic Approaches to Community Health (REACH) Charleston-Georgetown Diabetes Coalition

1. Approve meeting schedule that addresses the needs of its members, funding organizations, and community-based participatory approach groups, and review as needed.
2. Review mission, roles, membership, and guidelines annually.
3. Define goals and develop or update strategic plan to address goals annually.
4. Circulate and review minutes at the following meeting.
5. The chair and chair-elect create agendas 1 week in advance of each meeting and then review the agenda at the beginning of the meeting for any additions.
6. Invite board members to meetings with Centers for Disease Control and Prevention contacts as scheduled.
7. Prioritize communication between meetings. Contact the chair and chair-elect first and, if needed, contact the entire committee. Distribute notices for upcoming meetings and communications that need to occur between meetings by e-mail.
8. The chair and chair-elect communicate with members who have not attended at least half of the meetings to determine what about the coalition is and is not working for them, including their level of interest and commitment. Share feedback with the coalition and refine guidelines as needed.
9. REACH Community Action Plan teams from each member agency report in-depth on a rotating basis, and each team provides a short report.
10. Invite the liaison from each member agency to attend coalition meetings to report periodically on their projects.

### Appendix C. Example: Operating Principles — Racial and Ethnic Approaches to Community Health (REACH) Charleston-Georgetown Diabetes Coalition

To accomplish the REACH mission, principles guiding the conduct of projects and relationships are based on

- Building and sustaining effective partnerships for reducing or eliminating disparities.
- Establishing trust and building collaborative knowledge and understanding of the goals, objectives, and activities related to the problems (issues) we are addressing.
- Having an agreed-upon mission, values, goals, measurable outcomes, and accountability for the partnership.
- Building the relationships between partners including mutual trust, respect, genuineness, and commitment.
- Identifying strengths, assets, needs, and capacity of all partners.
- Balancing power among partners and enabling sharing of resources among partners.
- Having clear and open communication among partners while striving to understand each partner's needs and self-interests and while developing a common language.
- Establishing principles and processes for the partnership with the input and consensus of partners, especially for decision making and conflict resolution.
- Providing feedback among all stakeholders in the partnership, with the goal of continuously improving the partnership and its outcomes.
- Sharing the benefits of the partnership's accomplishments.
- Recognizing that a partnership can dissolve for multiple reasons but a planned process for closure is essential for all.
- Acknowledging accountability to sponsors and working collaboratively to reach requirements.
- Sharing ownership of and accountability to the grant and our program among all partners.
- Working together to sustain the benefits of collaboration and partnership.

These principles were based on principles of good community-campus partnerships (41). The coalition annually renews its partnership principles.

### Appendix D. Example: Community Advisory Board Leadership Structure — Sister-to-Sister Community and Academic Cochair Responsibilities

**Community cochair responsibilities**

1. Provide leadership to the Sister-to-Sister team in areas such as constituency engagement and communication, and creation of effective community and academic partnerships.
2. Lead board meetings every other month (alternate monthly meetings led by academic cochair).
3. Elicit agenda items from community residents and work with the academic cochair to establish the meeting agenda at least 2 weeks before the scheduled meeting date.
4. Ensure that meetings start and end at agreed-upon times.
5. Introduce each agenda item and facilitate round-robin discussion among all board members.
6. Elicit voting on key decisions, following the 70% rule of consensus.
7. Bring meetings to a conclusion with a summary of key issues decided on and any further follow-up that may be needed.
8. Coordinate the planned neighborhood activities guided by the study design.
9. Appoint ad hoc committees, as needed.
10. Represent the Sister-to-Sister team in discussions with community members and other networking forums as appropriate.
11. Monitor board members' attendance, participation, and ethical conduct as guided by the advisory board manual and operating procedures.
12. Monitor and make recommendations for the CAB supply budget.
13. Guide the evaluation process of study-related neighborhood activities and the CAB.

**Academic cochair responsibilities**

1. Provide leadership to the Sister-to-Sister team in areas such as research staff participation and communication, and creation of effective community and academic partnerships.
2. Lead board meetings (alternate meetings led by community cochair).
3. Elicit agenda items from the research team and work with the community cochair to establish meeting agenda at least 2 weeks before the scheduled meeting date.
4. Ensure the distribution of the agenda and previous meeting minutes (by mail) at least 1 week before the scheduled meeting date.
5. Ensure that meetings start and end at agreed-upon times.
6. Introduce each agenda item and facilitate round-robin discussion among all board members.
7. Elicit voting on key decisions, following the 70% rule of consensus.
8. Bring meetings to a conclusion with a summary of key issues decided on and any further follow-up that may be needed.
9. Work with the community cochair and assist with the coordination of the board's community activities as guided by the study.
10. Coordinate all technical support needed by the board and community events.
11. Represent the Sister-to-Sister team in discussions with community and academic members and other networking forums as appropriate.
12. Monitor board members' attendance, participation, and ethical conduct as guided by the advisory board manual and operating procedures.
13. Make logistical arrangements for food at meetings.
14. Monitor and process paperwork for CAB supplies and remuneration of community members.
15. Guide the evaluation process of study-related neighborhood activities and the CAB.

Source: Excerpt from the Sister-to-Sister Advisory Board Manual, 2008.

### Appendix E. Example: Evaluation Questions for Key Informant Interviews with Sister-to-Sister Neighborhood Advisory Board Members

1. Tell me about your experiences with the board so far.
2. Do you feel like the membership of the board reflects the community's interest? Is the community being represented in the way you think it should be?
3. Tell me about the meetings. Does everyone have the opportunity to present their opinions? How are the meetings conducted? How are conflicts resolved? Does anyone dominate the meetings? How are decisions made?
4. Do you have an understanding about the budget for the board's activities? Do you agree with how the resources are being used?
5. Do you think the board is accomplishing what it set out to do? What impact are the board's activities having on the community?
6. Are there any other challenges that you experienced on the board?
7. What are your recommendations for the board as we move forward with the project?
